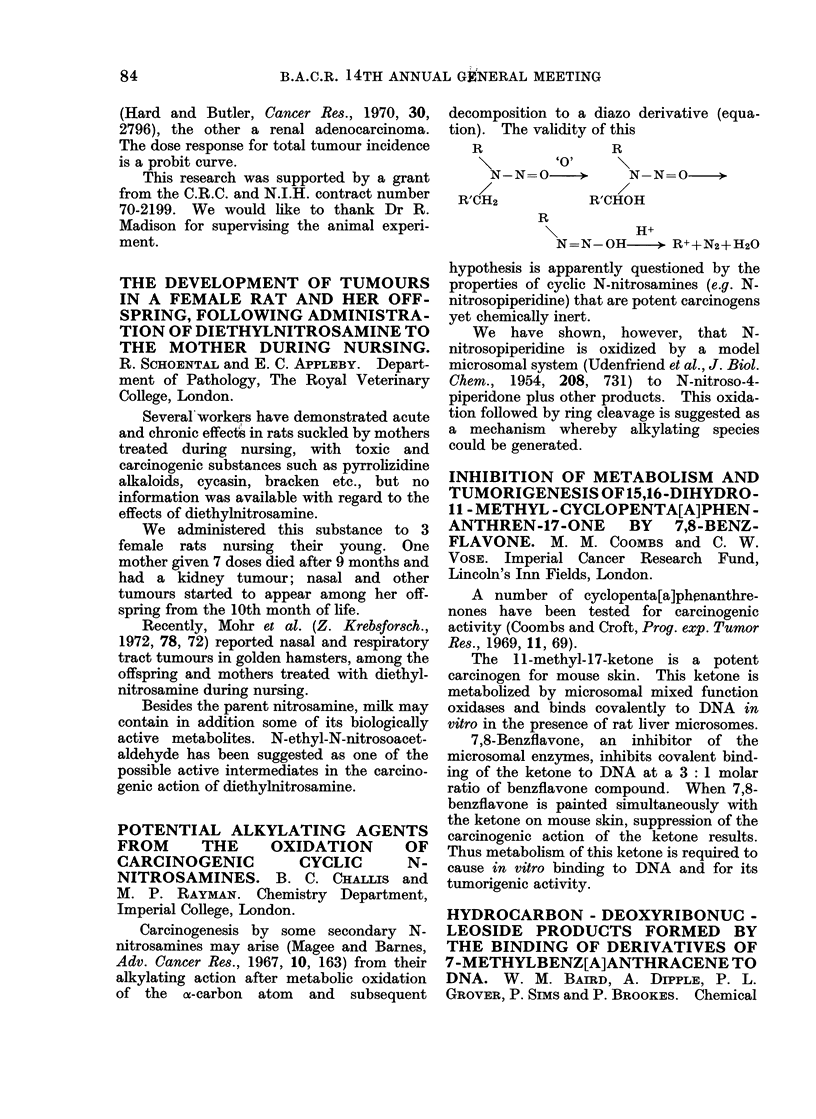# Inhibition of metabolism and tumorigenesis of 15,16-dihydro-11-methyl-cyclopenta(A)phenanthren-17-one by 7,8-Benzflavone.

**DOI:** 10.1038/bjc.1973.102

**Published:** 1973-07

**Authors:** M. M. Coombs, C. W. Vose


### INHIBITION OF METABOLISM AND TUMORIGENESIS OF 15,16-DIHYDRO-11-METHYL-CYCLOPENTA[A]PHENANTHREN-17-ONE BY 7,8-BENZFLAVONE.

M. M. Coombs and C. W. Vose. Imperial Cancer Research Fund, Lincoln's Inn Fields, London.

A number of cyclopenta[a]phenanthrenones have been tested for carcinogenic activity (Coombs and Croft, *Prog. exp. Tumor Res.*, 1969, **11**, 69).

The 11-methyl-17-ketone is a potent carcinogen for mouse skin. This ketone is metabolized by microsomal mixed function oxidases and binds covalently to DNA *in vitro* in the presence of rat liver microsomes.

7,8-Benzflavone, an inhibitor of the microsomal enzymes, inhibits covalent binding of the ketone to DNA at a 3 : 1 molar ratio of benzflavone compound. When 7,8-benzflavone is painted simultaneously with the ketone on mouse skin, suppression of the carcinogenic action of the ketone results. Thus metabolism of this ketone is required to cause *in vitro* binding to DNA and for its tumorigenic activity.